# Supplementary material for: Direct Photochemical Synthesis of Substituted Benzo[b]fluorenes
Source: Org Lett. 2024 Nov 25;26(48):10364–8. doi: 10.1021/acs.orglett.4c03978 (PMC11629382; doi:10.1021/acs.orglett.4c03978)
Supplement: Supplementary file 2 — ol4c03978_si_002.zip [file ol4c03978_si_002.zip › FID for Publication/5j'/20240615_RC-2-C-5-102-F44-48_01/plots/RC-2-C-5-102-F44-48_NOESY_20240615_01_plot01.pdf]

RC-2-C-5-102-F44-48

Location	8	Pulse sequence	NOESY	Temperature	25	Suboperator	RuairiCrawford
Date collected	2024-06-15	Solvent	cdcl3	Spectrometer	localhost.localdomain-vnmrs500	Operator	MBaumann

SAMPLE

date	Jun 15 2024
solvent	cdcl3
sample	20240615_RC-2-C-5-102-F44-48_01

ACQUISITION

sw	6097.6
at	0.150
np	1830
fb	4000
ss	32
d1	1.000
nt	8

2D ACQUISITION

sw1	6097.6
ni	256

TRANSMITTER

tn	H1
sfrq	499.722
tof	13.1
tpwr	58
pw	6.300

NOESY

mixN	0.500

PRESATURATION

satmode	n
wet	n

DECOUPLER

dn	C13
dm	nnn

FLAGS

hs	nn
sspul	y
PFGflg	y
hsglvl	5736

SPECIAL

temp	25.0
gain	46
spin	0

F2 PROCESSING

gf	0.069
gfs	not used
fn	2048

F1 PROCESSING

gf1	0.029
gfs1	not used
proc1	lp
fn1	2048

DISPLAY

sp	-537.1
wp	6097.6
sp1	-537.1
wp1	6097.6
rfl	537.1
rfp	0
rfl1	537.1
rfp1	0

PLOT

wc	142.6
sc	0
wc2	127.1
sc2	12.7
vs	117
th	3
ai	ph

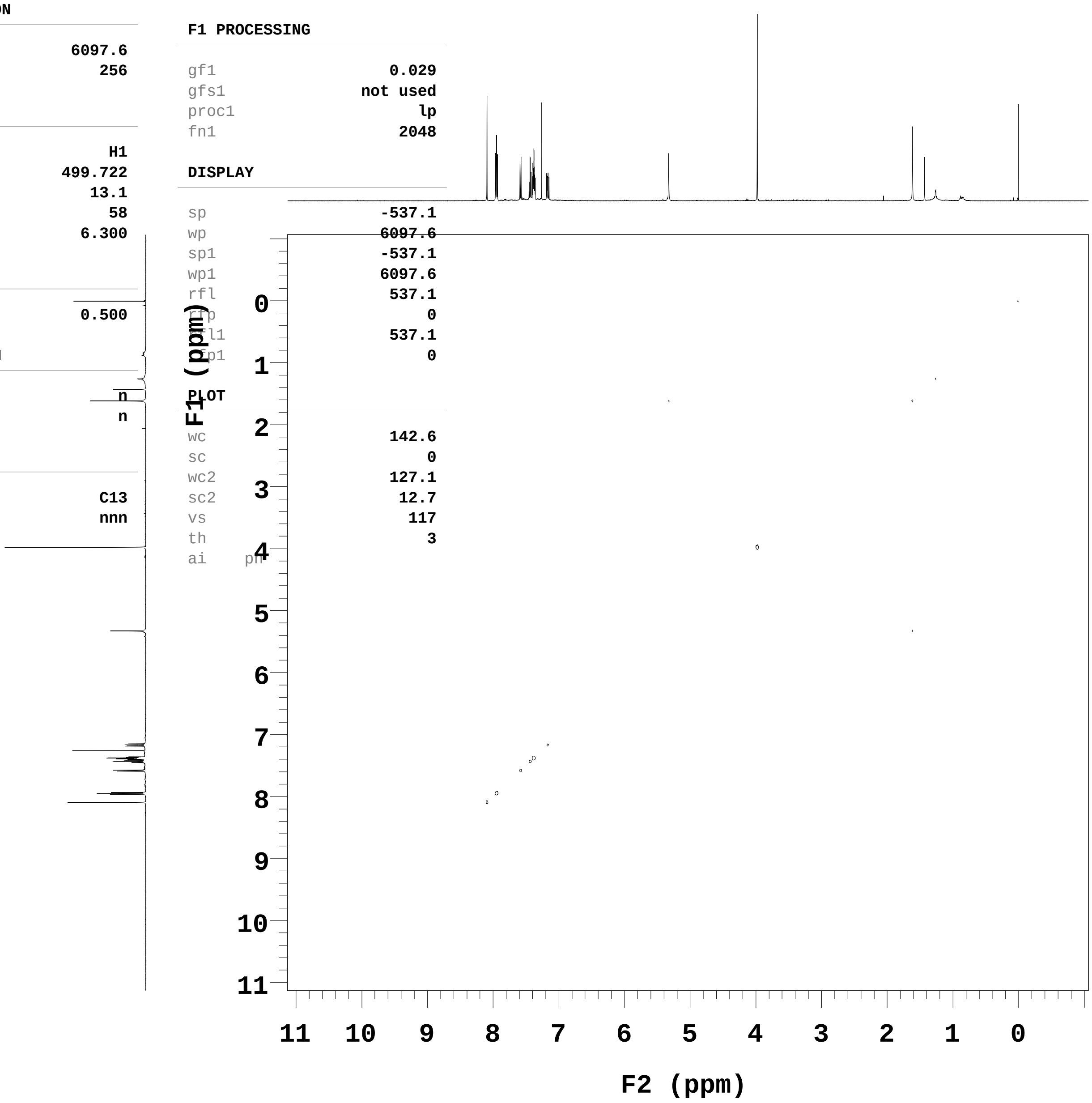

Plotname: RC-2-C-5-102-F44-48_NOESY_20240615_01_plot01

Data file /home/data/MBaumann/cscb500/RuairiCrawford/2024/20240615_RC-2-C-5-102-F44-48_01/RC-2-C-5-102-F44-48_NOESY_20240615_01.fid
